# The vicious cycle of BRASH syndrome: A case report

**DOI:** 10.21542/gcsp.2023.2

**Published:** 2023-01-30

**Authors:** Twinkle Saini, Jacky Reny, Hussam Al Hennawi, Andrew Cox, Chaitra Janga, Danila DeLiana, James McCaffrey

**Affiliations:** 1 Philadelphia College of Osteopathic Medicine, Philadelphia, PA, USA; 2 Thomas Jefferson University, Philadelphia, PA, USA; 3 Department of Internal Medicine, Jefferson Abington Hospital, Abington, PA, USA

## Abstract

First described in 2016, BRASH syndrome is an underreported clinical entity characterized by bradycardia, renal dysfunction, atrioventricular nodal blockade (AVNB), shock, and hyperkalemia. The recognition of BRASH syndrome as a clinical entity is crucial for early and effective management. Patients with BRASH syndrome present with symptomatic bradycardia that is resistant to treatment with standard agents such as atropine. In this report, we present the case of a 67-year-old male patient who presented with symptomatic bradycardia with an ultimate diagnosis of BRASH syndrome. We also shed light on predisposing factors and challenges encountered during the management of affected patients.

## Introduction

BRASH is an acronym used to describe a syndrome arising from a combination of renal insufficiency and negative chronotropic and/or inotropic agents. The sequelae of the signs composing the recently described entity include bradycardia, renal failure, AV nodal blockade, shock, and hyperkalemia. The pathophysiology of BRASH syndrome is derived from synergism between AV nodal blockade and hyperkalemia. A seemingly innocuous event such as mild dehydration causes a mild reduction in renal perfusion and reduced GFR. Renally cleared cardiovascular medications such as beta-blockers, non-dihydropyridine calcium channel blockers, and antiarrhythmics begin to accumulate due to this insult^1^.

**Figure 1.**
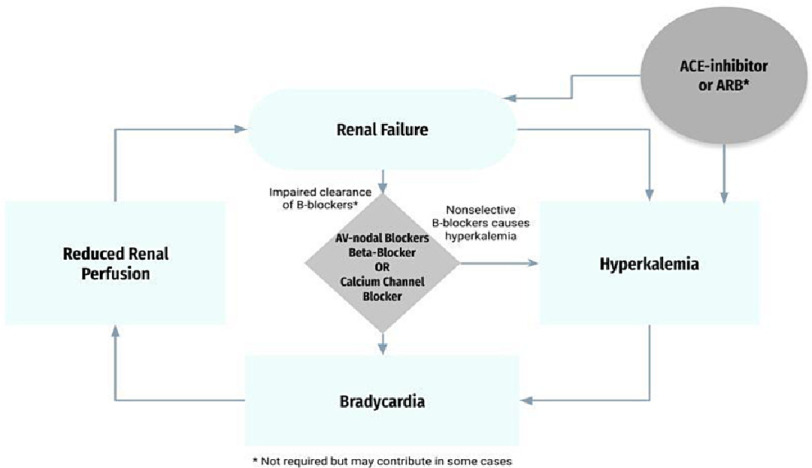
BRASH syndrome pathophysiology.

Continued accumulation results in bradycardia and decreased cardiac output, which induces renal hypoperfusion, precipitating compounding renal failure^2^. Renal failure can induce hyperkalemia and further decrease the excretion of drugs that are partially or fully renally cleared (Figure 1). Therapeutic doses of such agents generally do not cause severe bradycardia; however, with decreased renal clearance and hyperkalemia, the effects of AV nodal blockers are potentiated. Failure to recognize this cycle contributes to poor management, and upon discharge, resuming previously prescribed medications may result in repeated hospitalizations and unnecessary interventions. AV nodal blockers have been used widely to treat a range of conditions, such as hypertension and arrhythmia, but the recognition and characterization of BRASH as its own entity is rather novel.

While the current prevalence of BRASH has not been entirely elucidated, with the aging population and the growing standard of more aggressive preventative care, such as more restrictive blood pressure management, there is an increased need to characterize the unique presentations and management of BRASH syndrome^3^. The following case presentation includes two hospital visits for BRASH syndrome, for which the patient was managed with varying approaches.

### Case presentation

A 67-year-old male presented with new-onset fatigue and lightheadedness while golfing. His heart rate at home was 34 bpm, which prompted him to visit the emergency (ED) for evaluation. He denied angina, syncope, or palpitations. He had no recent medication changes and had a strong history of medication compliance. His past medical history was significant for atrial fibrillation status-post ablation 8 years prior on rhythm control with flecainide, type-one diabetes on an insulin pump, obstructive sleep apnea, and chronic kidney disease stage IIIb. He had undergone echocardiogram one month prior, which showed an ejection fraction of 60–65% with mild left ventricular hypertrophy, grade one diastolic dysfunction, and no valvular disease. The results of the outpatient nuclear medicine stress test were unremarkable. His outpatient medication regimen included apixaban, amlodipine, flecainide, simvastatin, hydrochlorothiazide, lisinopril, metoprolol, and insulin. Renally cleared medications were administered at recommended ranges for glomerular filtration rate (GFR).

On arrival, his vital signs were significant, with a heart rate of 24 bpm, blood pressure of 142/66 mmHg, respiratory rate 18/min, saturating of 99% on room air, and body temperature of 36 °C. Labs were significant for potassium 5.6 mmol/L, Cr 2.12 mg/dL (baseline 1.7), BUN 43 mg/dL, AST 126 IU/L, ALT 147 IU/L, Hgb 17.1 g/dL, and initial troponin of 15 ng/L while all other laboratory results were within normal limits. Electrocardiography (EKG) showed sinus bradycardia with no evidence of heart block or signs of ischemia. Chest radiography revealed no evidence of active disease. Hyperkalemia was managed using insulin, dextrose, and sodium polystyrene sulfonate. Possible differential diagnoses include medication-induced bradycardia and BRASH syndrome. Owing to symptomatic bradycardia, his home metoprolol succinate and flecainide were held. In the setting of acute kidney injury, lisinopril and hydrochlorothiazide were also withheld, along with intravenous fluids. Over the next twenty-four hours his bradycardia and hyperkalemia resolved and the patient was discharged from the ED on a reduced dose of metoprolol succinate with instructions to resume flecainide 3 days after discharge. The remainder of his home medications did not change.

**Figure 2.**
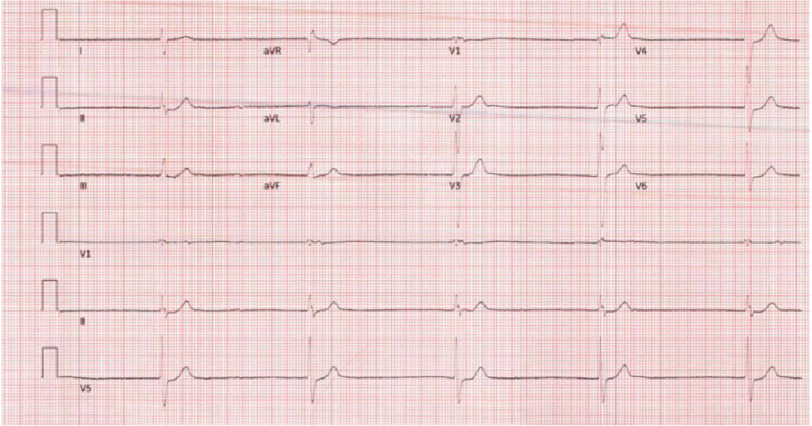
Initial EKG showing bradycardia with a rate of 30 and complete heart block with ventricular escape beats. Note the absence of EKG changes suggestive of severe hyperkalemia that can cause similar bradyarrhythmia.

Three months later, he presented again with similar complaints of fatigue after golfing. He also reported dizziness, generalized weakness, nausea, vomiting, and diaphoresis. In the ED, heart rate was 35 bpm, blood pressure was 167/71 mmHg, respiratory rate was 20, and saturation was 95% on room air, with a temperature of 98.4°F. Physical examination revealed bradycardia with an irregular rhythm and cold extremities upon touch. Initial laboratory work showed high potassium levels (6.2 mmol/L), and elevated creatinine levels (2.77 mg/dL, baseline 1.7). His remaining laboratory work was insignificant, except for a mild elevation in troponin levels to 18 (normal < 6). EKG confirmed bradycardia at a rate of 30 with complete heart block and ventricular escape complexes (Figure 2). Chest radiography revealed no acute cardiopulmonary abnormality. The patient was administered fluids for acute kidney injury (AKI) and hyperkalemia was managed with calcium gluconate, insulin, furosemide, and albuterol.

**Figure 3.**
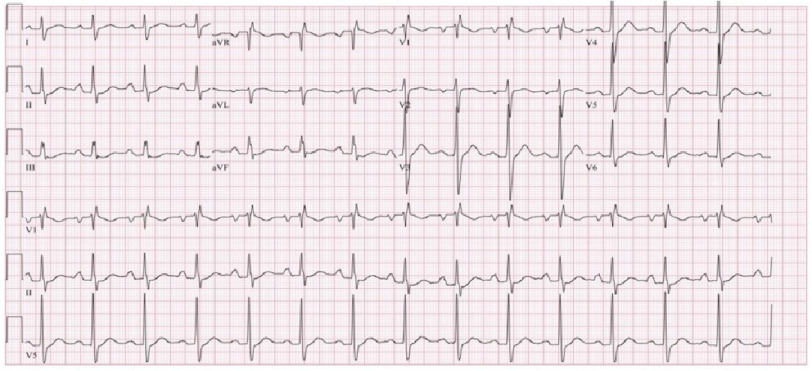
Outpatient follow-up electrocardiogram showing normal sinus rhythm with a heart rate of 83 bpm.

During this admission, the differential diagnosis of BRASH syndrome was high; hence, both flecainide and metoprolol were discontinued. He reverted to sinus rhythm after taking these medications and maintained the rates in the 50s following interventions. AKI was resolved with fluids, and potassium normalized to 3.9 mmol/L on discharge with discontinuation of lisinopril. He was discharged on amlodipine and hydrochlorothiazide for hypertension, with plans to hold flecainide, metoprolol, and lisinopril indefinitely due to the risks, which were now apparent. In the follow-up labs, his potassium level remained within normal limits and sustained improvement in heart rate without high-grade atrioventricular (AV) block (Figure 3).

## Discussion

BRASH syndrome is a combination of impaired renal clearance that causes hyperkalemia and build-up of AV nodal blocking medications, leading to bradycardia and shock^4^. Impaired renal function leads to the buildup of AV nodal blocking agents, because most agents are renally cleared. Hyperkalemia and AV nodal blocking agents do not usually cause this degree of bradycardia, except for severe hyperkalemia or intentional overdose^5^. Bradycardia leads to decreased cardiac output, decreased renal perfusion, and acute kidney injury, which worsens or induces hyperkalemia^6^. Renal clearance impairment leads to further accumulation of AV nodal blocking agents, thus contributing to severe bradycardia^5^. This cycle of renal insufficiency and bradycardia can progress to shock and subsequent multiple-organ failure^6^.

In BRASH syndrome, management of hyperkalemia is very important^5^. Rapid management of hyperkalemia can be achieved through the administration of IV calcium to stabilize the cardiac membrane and improve cardiac output; however, it does not reduce the extracellular potassium concentration^7^. Intravenous insulin and dextrose were used to intracellularly shift potassium. Beta-agonists work similarly, but when given via their typical route (i.e., inhaled short-acting beta agonists), are less efficacious.

Sodium polystyrene sulfonate is often used to reduce total body potassium. Sodium polystyrene sulfonate binds to potassium within the colon in exchange for sodium, and potassium is then excreted^7^. Sodium zirconium cyclosilicate has a mechanism similar that to of sodium polystyrene sulfonate, but has a shorter time of action^6^. Another potassium dumping agent is furosemide, which removes potassium via renal excretion^7^.

On the first admission of our patient, one dose of IV insulin, dextrose, calcium gluconate, and sodium polystyrene sulfonate was administered. This lowered the potassium from 5.6 to 4.2 mmol/L on discharge. On the second admission, albuterol, calcium gluconate, insulin, dextrose, furosemide, and sodium zirconium cyclosilicate were administered. This lowered the potassium from 6.2 to 5.3 mmol/L on discharge. During follow-up, potassium was rechecked twice as an outpatient at normal levels. Another aspect of BRASH syndrome management is the hemodynamic support for bradycardia and hypotension^5^. Hypovolemic patients should receive lactated Ringer’s solution or, if acidotic, isotonic bicarbonate^6^. High-volume administration of normal saline can theoretically induce temporary hyperchloremic metabolic acidosis and shift potassium extracellularly, worsening hyperkalemia and making this a poor choice for volume resuscitation^6^.

Finally, recognition and adjustment of the triggering agent are key^5^. At the first admission, both metoprolol and flecainide were withheld. After seeing that his heart rate was stabilized, flecainide was restarted and home metoprolol was halved. The patient followed up with his primary care provider as an outpatient, his heart rate was low-to-normal, and the patient refused further tests at the time.

During the initial hospitalization, cardiology acknowledged that renal impairment caused the accumulation of flecainide and metoprolol; however, metoprolol was the only medication that was changed. This is likely due to the well-known effect of metoprolol on bradycardia. In the second encounter, both flecainide and metoprolol were discontinued, acknowledging the effect of renal insufficiency in the accumulation of these medications leading to bradycardia and hyperkalemia. After this change, the patient maintained sinus rhythm at appropriate rates and did not present to the hospital for symptomatic bradycardia.

### What have we learned?

In this case of BRASH syndrome, the patient was admitted twice within a couple of months for the same reason. On the initial hospital visit, BRASH syndrome was suspected; however, only metoprolol was changed, and flecainide was continued after being withheld during hospitalization. In the second encounter, both medications were discontinued acknowledging the effects of renal insufficiency on increasing circulating metoprolol and flecainide. The patient had been taking these medications without issue for many years, but due to the progression of chronic kidney disease, the pharmacokinetic profile of his medications had been changing unrecognized by his providers. Recognition of BRASH syndrome as a relatively common and distinct entity of iatrogenic origin is important for providers to make appropriate decisions for medications prescribed in the large population of patients with renal insufficiency and cardiovascular disease.
